# High-Quality Reference Genome Sequence for the Oomycete Vegetable Pathogen Phytophthora capsici Strain LT1534

**DOI:** 10.1128/MRA.00295-21

**Published:** 2021-05-27

**Authors:** Jason E. Stajich, Andrea L. Vu, Howard S. Judelson, Gregory M. Vogel, Michael A. Gore, Maryn O. Carlson, Nicholas Devitt, Jennifer Jacobi, Joann Mudge, Kurt H. Lamour, Christine D. Smart

**Affiliations:** a Department of Microbiology and Plant Pathology, University of California, Riverside, California, USA; b Institute for Integrative Genome Biology, University of California, Riverside, California, USA; c School of Integrative Plant Science, Cornell University, Geneva, New York, USA; d National Center for Genome Resources, Santa Fe, New Mexico, USA; e Department of Entomology and Plant Pathology, University of Tennessee, Knoxville, Tennessee, USA; Vanderbilt University

## Abstract

The oomycete Phytophthora capsici is a destructive pathogen of a wide range of vegetable hosts, especially peppers and cucurbits. A 94.17-Mb genome assembly was constructed using PacBio and Illumina data and annotated with support from transcriptome sequencing (RNA-Seq) reads.

## ANNOUNCEMENT

**FIG 1:**
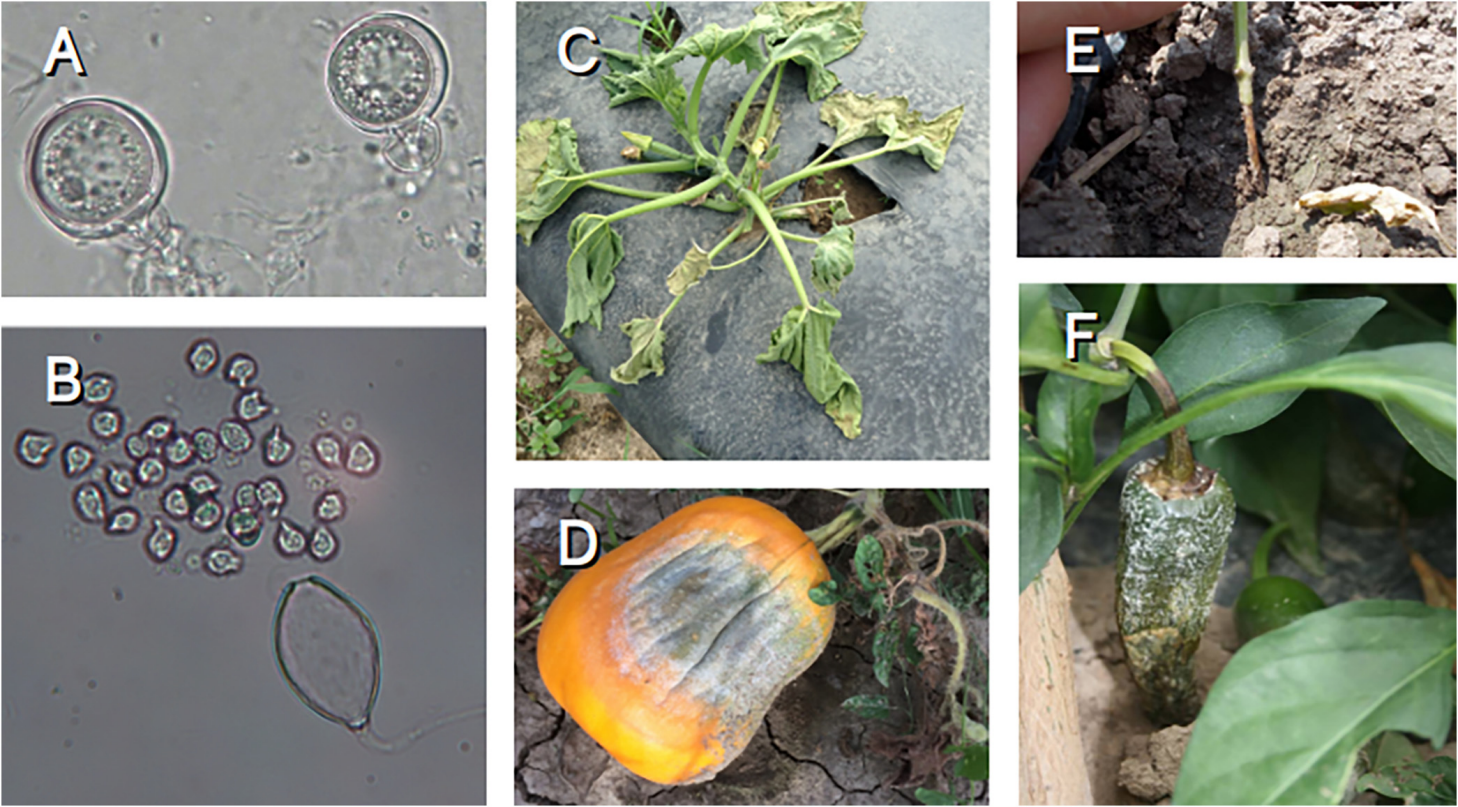
Micrographs of *P. capsici* reproductive structures and examples of disease symptoms. (A) Oospores, the sexual overwintering structures, with amphigynous antheridia in view. (B) A sporangium releasing motile zoospores. The sporangiophore can be seen attached to the base of the sporangium. (C) Crown rot on zucchini causing total plant wilt and death. (D) Fruit rot on pumpkin with visible white sporangia. (E) Stem of young pepper plant showing brown girdling due to crown rot. (F) Fruit rot on jalapeno pepper. Symptoms started at the tip of the fruit, which is touching the soil, and moved up the fruit. Typical white symptoms are sporangia of *P. capsici* with each sporangium on a sporangiophore. There would be thousands of sporangia per infected fruit similar to the one shown here.

Phytophthora capsici is a highly destructive pathogen of vegetables worldwide, especially those in the Solanaceae and Cucurbitaceae families (Fig. 1). Efforts to understand the role of genes in pathogenesis and host range (1–8), population structure (9, 10), genomic variation (11), and development (12) benefit from an accurate genome assembly. *P. capsici* belongs to the eukaryotic phylum Oomycota, many members of which have repeat-rich genomes that are difficult to assemble with short reads. Strain LT1534, A2 mating type, is an inbred strain derived from crossing isolates from infected cucurbits in Michigan (cucumber) and Tennessee (pumpkin) (11). An earlier assembly for LT1534, generated with Roche 454 and Sanger sequencing (11), was 64 Mb in 917 scaffolds (*N*_50_, 706 kb).

A single oospore-derived isolate (LT1534) was maintained axenically on PARP (25 ppm pimaricin, 100 ppm ampicillin, 25 ppm rifampicin, and 100 ppm pentachloronitrobenzene) agar plates, and a small weft of mycelium was transferred to clarified V8 juice broth amended with PARP and grown at 22 to 23°C for 7 days prior to harvesting, freeze-drying, disruption, and extraction of genomic DNA (13) using a GeneJet genomic DNA purification kit (Thermo Fisher). Libraries were constructed with a TruSeq DNA kit and sequenced on an Illumina HiSeq X Ten system by Novogene (Shenzen, China), yielding 34.9 million, 2 × 150-bp read pairs (10.48 Gbp). PacBio libraries were constructed by the National Center for Genome Resources (Santa Fe, NM) following the 20-kb protocol (14). One single-molecule real-time (SMRT) cell was sequenced on a PacBio RS II system using P6 polymerase and C4 chemistry, yielding 964,374 subreads (*n* = 964,374; *N*_50_, 12.5 kb; maximum, 47.5 kb; total, 8.15 Gbp) as processed by the SMRT pipeline v2.3.0.1. PacBio reads were corrected with Illumina by LoRDEC v0.9 (15) using the default parameters.

To provide gene model support, mRNA was extracted from 2.5-, 3-, and 4.5-day V8 agar cultures grown at 23°C under 12-h light/dark conditions using a Spectrum plant total RNA kit (Sigma). Strand-specific transcriptome sequencing (RNA-Seq) libraries were constructed and sequenced by Cofactor Genomics (St. Louis, MO) using oligo(dT) priming on an Illumina NextSeq 500 instrument to obtain ∼25 million single-end reads per library.

Read trimming, correction, and genome assembly were performed with MaSuRCA v3.3.1 (16, 17) with Illumina and LoRDEC-corrected PacBio reads (LHE_COVERAGE=25 cgwErrorRate=0.15). The assembly was screened for adaptors and contamination using AAFTF v0.2.3 (18). Assembly polishing with masurca-polish.sh corrected 1,025 substitutions and 2,643 indels, resulting in 99.9961% computed consensus quality. The 94.17-Mb assembly is in 782 scaffolds (*N*_50_, 485 kb; *L*_50_, 44; average GC content, 52.3%), and the longest scaffold covers 4.61 Mb. Completeness was assessed with BUSCO v3.0.2 (19, 20) using protists_ensembl v9, resulting in the identification of 211 (98.1%) out of 215 genes; 175 were single-copy complete, 36 were duplicated, and 2 were fragmented.

Genome annotation was performed with Funannotate v1.8.1 (21) using default parameters, which implemented the following steps. Prediction training and annotation were supported by RNA-Seq reads aligned to the genome with HISAT2 v2.2.1 (22) and reference-guided transcript assembly in Trinity v2.11.0 (maxintron=4kb) (23, 24) and PASA v2.4.1 (25). The best gene models were used to train and run SNAP v2013_11_29 (26) and AUGUSTUS v3.3.3 (27). Additional *ab initio* models were predicted using GeneMark v4.59 (28), GlimmerHMM v3.0.4 (29), and CodingQuarry v2.0 (30). Evidence for exons was generated by DIAMOND v2.0.4 (31) and Exonerate v2.4.0 (32) alignments of SwissprotDB (33) proteins. Consensus gene models were produced with EVidenceModeler v1.1.1 (25) using Funannotate default evidence weights. Untranslated regions and alternatively spliced isoforms were predicted using PASA from RNA-Seq. The putative protein function was assigned by sequence similarity to the InterProScan v5.45-80.0 (34), eggNOG v1.0.3 (35), dbCAN2 v9.0 (36), and MEROPS v12.0 (37) databases. The genome has 23,373 predicted protein-coding genes, 133 of which had at least one putatively alternatively spliced isoform.

### Data availability.

This whole-genome shotgun project has been deposited at DDBJ/ENA/GenBank as accession number JADEVP000000000. The version described in this paper is version JADEVP010000000. The PacBio (SRA number SRR13176613) and Illumina (SRA number SRR13176614) genomic sequencing reads are associated with BioProject PRJNA481983. The RNA-Seq reads are associated with BioProject PRJNA692306 and deposited under SRA project SRP301859 (SRA numbers SRR13441373 to SRR13441375).
